# Reduced Endothelial Function in Myalgic Encephalomyelitis/Chronic Fatigue Syndrome–Results From Open-Label Cyclophosphamide Intervention Study

**DOI:** 10.3389/fmed.2021.642710

**Published:** 2021-03-22

**Authors:** Kari Sørland, Miriam Kristine Sandvik, Ingrid Gurvin Rekeland, Lis Ribu, Milada Cvancarova Småstuen, Olav Mella, Øystein Fluge

**Affiliations:** ^1^Department of Oncology and Medical Physics, Haukeland University Hospital, Bergen, Norway; ^2^Faculty of Health Sciences, Oslo Metropolitan University, Oslo, Norway; ^3^Porsgrunn District Psychiatric Centre, Telemark Hospital Trust, Porsgrunn, Norway; ^4^Department of Clinical Science, Institute of Medicine, University of Bergen, Bergen, Norway

**Keywords:** myalgic encephalomyelitis, chronic fatigue syndrome, ME/CFS, endothelial function, flow-mediated dilation, post-occlusive reactive hyperemia, cyclophosphamide

## Abstract

**Introduction:** Patients with myalgic encephalomyelitis/chronic fatigue syndrome (ME/CFS) present with a range of symptoms including post-exertional malaise (PEM), orthostatic intolerance, and autonomic dysfunction. Dysfunction of the blood vessel endothelium could be an underlying biological mechanism, resulting in inability to fine-tune regulation of blood flow according to the metabolic demands of tissues. The objectives of the present study were to investigate endothelial function in ME/CFS patients compared to healthy individuals, and assess possible changes in endothelial function after intervention with IV cyclophosphamide.

**Methods:** This substudy to the open-label phase II trial “Cyclophosphamide in ME/CFS” included 40 patients with mild-moderate to severe ME/CFS according to Canadian consensus criteria, aged 18–65 years. Endothelial function was measured by Flow-mediated dilation (FMD) and Post-occlusive reactive hyperemia (PORH) at baseline and repeated after 12 months. Endothelial function at baseline was compared with two cohorts of healthy controls (*N* = 66 and *N* = 30) from previous studies. Changes in endothelial function after 12 months were assessed and correlated with clinical response to cyclophosphamide. Biological markers for endothelial function were measured in serum at baseline and compared with healthy controls (*N* = 30).

**Results:** Baseline FMD was significantly reduced in patients (median FMD 5.9%, range 0.5–13.1, *n* = 35) compared to healthy individuals (median FMD 7.7%, range 0.7–21, *n* = 66) (*p* = 0.005), as was PORH with patient score median 1,331 p.u. (range 343–4,334) vs. healthy individuals 1,886 p.u. (range 808–8,158) (*p* = 0.003). No significant associations were found between clinical response to cyclophosphamide intervention (reported in 55% of patients) and changes in FMD/PORH from baseline to 12 months. Serum levels of metabolites associated with endothelial dysfunction showed no significant differences between ME/CFS patients and healthy controls.

**Conclusions:** Patients with ME/CFS had reduced endothelial function affecting both large and small vessels compared to healthy controls. Changes in endothelial function did not follow clinical responses during follow-up after cyclophosphamide IV intervention.

## Introduction

Myalgic Encephalomyelitis/Chronic Fatigue Syndrome (ME/CFS) is a disease that affects both children and adults and is associated with very low health-related quality of life (1, 2). Patients present with hallmark symptoms such as post-exertional malaise (PEM), fatigue and lack of adequate restitution from rest or sleep, accompanied by pain, cognitive symptoms and sensory hypersensitivity (3, 4). Prevalence of ME/CFS as diagnosed by the Canadian consensus criteria (4) is estimated in the United Kingdom in primary health care at 0.1 to 0.2 per cent of the population (5). A report commissioned by the US Institute of Medicine concluded that ME/CFS is a serious, systemic disease with no known cause or cure, and that there is a great need for research to further understanding of the disease (6). There is a scarcity of research evidence and the disease mechanisms are poorly understood (7).

Symptoms frequently present following an event such as infection, physical trauma or exposure to environmental factors (4), and there is evidence of a genetic predisposition (8, 9). Research suggests the involvement of the immune system (10, 11), an impaired energy metabolism (12–14) and alterations in the gut microbiome (15). Orthostatic intolerance and autonomic dysfunction are also frequently reported, and can present with symptoms such as light-headedness, nausea, concentration difficulties, sweating, palpitations, dyspnea, and chest pain after prolonged sitting or standing (6).

It has been suggested that a dysfunction of the blood vessel endothelium could be a contributing mechanism, possibly associated with inadequate fine-tuned regulation of blood flow according to the metabolic demands of tissues (16, 17). One possible explanation for such endothelial dysfunction could be a reduced bioavailability of nitric oxide (NO) derived from endothelial cells. NO is a messenger molecule and neurotransmitter with important effects on vasodilation, thus contributing to the regulation of blood flow to tissues. NO is involved in many biologic processes, including effects on cognitive function, smooth muscle tone in the gastrointestinal and urogenital tracts, cardiac contractility, skeletal muscle and mitochondrial function (18, 19). Non-invasive measures for endothelial function include *flow-mediated dilation* (FMD) of the large arteries, which is believed to reflect the release of NO from endothelial cells caused by shear stress in vessel walls (20–22). As part of an FMD investigation, it is recommended to also measure endothelium-independent vasodilation, reflecting smooth muscle function, through the administration of sublingual nitroglycerin (20, 21). In the microcirculation, the measure *post-occlusive reactive hyperemia* (PORH) is understood to represent a more complex response involving nervous and myogenic responses as well as several vasodilators including NO (23).

There is growing evidence for endothelial dysfunction in autoimmune diseases (20, 24, 25) and fibromyalgia (26). However, research into blood vessel function in ME/CFS is limited so far. A study by Newton and colleagues (16) showed reduced FMD and PORH in a group of ME/CFS patients compared to healthy controls. This finding was confirmed by Scherbakov et al. who reported peripheral endothelial dysfunction in 18 of 35 (51%) ME/CFS patients compared to 4 of 20 (20%) healthy controls. This study also indicated a correlation between endothelial dysfunction and disease severity (17). In contrast, another study of 24 ME/CFS patients and 24 sedentary controls, using a different assessment method, found no significant difference in peripheral endothelial function at rest or after exercise (27).

In 2014, we performed a small pilot study at Haukeland University Hospital and found very low values of FMD in ME/CFS patients (unpublished). Following the pilot experience, studies of endothelial function were performed as substudies to two intervention drug trials for patients with ME/CFS at Haukeland University Hospital: a phase III trial of rituximab vs. placebo (28) and a phase II trial of cyclophosphamide (29). This article reports the results from the endothelial function substudy of the cyclophosphamide trial.

The purpose of this study was to explore: (1) endothelial function, measured by flow-mediated dilation (FMD) and post-occlusive reactive hyperemia (PORH), in a Norwegian cohort of ME/CFS patients compared with healthy individuals, (2) changes in FMD and PORH from baseline to 12 months, and possible associations between clinical response to cyclophosphamide and changes in FMD/PORH over time.

## Methods

### Design

This study was performed as a substudy to the open-label phase II trial “Cyclophosphamide in myalgic encephalomyelitis/chronic fatigue syndrome” (29). The study has a cross-sectional design, comparing endothelial function in patients at baseline with healthy individuals. In addition, the study has a longitudinal element, which explores associations between clinical response after cyclophosphamide intervention and changes in endothelial function for the patient group over time.

### Setting

Patients were recruited from March to December 2015 at Haukeland University Hospital in Bergen, Norway. Baseline measurements were performed successively after inclusion in the clinical trial (March 2015–January 2016), followed by intervention with six infusions of cyclophosphamide IV 4 weeks apart. Measurements were repeated 12 months after inclusion in the trial (March 2016–January 2017).

### Patient Inclusion

#### Patient Group

The 40 patients included in this study were all enrolled in the open-label phase II clinical trial *Cyclophosphamide in ME/CFS*. Inclusion criteria were age 18 to 65, a confirmed ME/CFS diagnosis according to Canadian consensus criteria (4), disease duration of minimum 2 years, disease severity from mild-moderate to severe (excluding mild and very severe ME/CFS) and signed informed consent. Following the publication of previous intervention trials (30, 31), we received referrals from general practitioners and applications directly from patients, requesting evaluation for any future clinical trials. After a preliminary assessment of eligibility based on records of medical history and current disease severity, we performed a random selection among candidates who met the inclusion criteria. Candidates were invited to receive further information on the trial and this substudy, and subject to informed consent, they were screened according to protocol (29). Fifteen patients were recruited among previous rituximab trial participants (non-responders or responders with full or partial relapse) (30, 31).

#### Healthy Individuals

Reference baseline values for FMD and PORH for healthy individuals were obtained from two other studies performed by the authors and using the same protocols for measurement of endothelial function as those employed in this study. The reference group for FMD consisted of 66 healthy controls participating in a study of endothelial function in women with pre-eclampsia performed by MKS (32), and the reference group for PORH consisted of 30 healthy volunteers examined by KS for an endothelial function substudy to the multi-center RCT *RituxME* (28). The FMD reference group was all-female, while the gender distribution of the PORH reference group was similar to that of the patient group. There were no significant differences between the patient and reference groups with regards to age, BMI, resting blood pressure or heart rate.

### Intervention

Patients were scheduled to receive medical intervention with six intravenous infusions of cyclophosphamide (initial dosage of 600 mg/m^2^, increased to 700 mg/m^2^ for the following five infusions conditional on acceptable hematological toxicity). Nine patients deviated from the treatment protocol and received from 3 to 5 infusions. During the trial, 22 of 40 patients met the criteria for clinical response, defined as Fatigue Score ≥4.5 for a minimum of 6 consecutive weeks. SF-36 Physical Function scores among the 22 responders increased from mean 35.0 to 69.5 points (scale 0–100). The median response duration in 18 months follow-up was 44 weeks, and among responders the majority had prolonged remission for years. See Rekeland et al. (29) for details on the trial schedule and results.

### Outcomes

Main outcomes were measures of endothelial function—flow-mediated dilation and post-occlusive reactive hyperemia—at baseline and at 12 months after inclusion in the trial. Variables included in the statistical analyses included clinical response status, disease severity and other clinical variables, as well as biological markers of endothelial function.

#### Measurement of FMD and PORH

Measurements were performed and reported according to a standardized protocol following guidelines from the International Brachial Artery Reactivity Task Force (21). Subject preparations and measurement procedures are described in detail in the study protocol (Supplementary Material). Flow-mediated dilation (FMD) of the brachial artery was measured using a GE Vingmed Vivid E9 ultrasound system (GE Vingmed Ultrasound, Horten, Norway) with a multifrequency linear probe, 6–13 mHz (M12L). Participants were instructed to fast with regards to food, fluids (except water), tobacco and medicines for 8 h before assessment. The assessments took place in a quiet, dark, and temperature-controlled room, where participants relaxed on the examination table for at least 10 min pre-assessment. The brachial artery was imaged in the longitudinal plane above the antecubital fossa and a baseline rest image was acquired. A blood pressure cuff, positioned on the forearm, was inflated to 200 mmHg or at least 50 mmHg above above systolic pressure, for 5 min. Following deflation of the cuff, images were recorded continuously from the same area of the artery during the next 5 min. All measurements were performed during end diastole. FMD is expressed as diameter increase in per cent from baseline to time of maximum dilation. After rest, a dose of nitroglycerine spray (0.4 mg) was administered sublingually, and images were recorded continuously for another 5 min. The maximal diameter was measured to express endothelium independent vasodilation.

During the same procedure, measurements of microvascular function (post-occlusive reactive hyperemia, PORH) were performed using a Periflux 5000 laser Doppler unit (Perimed, Stockholm, Sweden). A temperature-controlled probe was placed on clean, intact forearm skin and microvascular perfusion was recorded before and after occlusion. PORH is expressed in perfusion units (PUs) as the difference (area under the curve) between circulation in the skin during 2 min at baseline and the first 2 min after cuff deflation.

FMD and PORH measurements were performed by KS. All ultrasound images were analyzed by KS. In addition, M.S. analyzed a randomly selected 10 % of the ultrasound measurements for FMD (*n* = 10), one of which had questionable image quality and was excluded from analyses. Inter-observer variability for the remaining measurements was computed using a two-way mixed effects, absolute agreement, single measures model (33, 34). The intraclass correlation coefficient was excellent for baseline measurements of artery diameter (ICC = 0.99) and good for FMD (ICC = 0.77).

#### Clinical Response

Every 2 weeks during 18 months follow-up, patients were requested to complete a self-report form to capture the relative change in symptoms from baseline to the time of recording. The scale for symptom change was adapted from the Clinical Global Impression Scale which had previously been used in studies of ME/CFS (35), and has been employed in the follow-up of patients in previous clinical trials (28, 30, 31, 36). The scale for each symptom was 0 to 6, where 0 denoted major worsening, and 6 major improvement of the symptom compared to baseline. The variable Fatigue Score was calculated as the mean change score for four fatigue-related items: “Fatigue,” “Post-exertional malaise,” “Need for rest” and “Daily functioning.” Clinical response was defined as Fatigue Score ≥4.5 for at least 6 consecutive weeks (29).

#### Clinical and Sociodemographic Variables

Before inclusion in the cyclophosphamide trial, participants were subject to a clinical examination/interview. Clinical variables reported are age, sex, disease severity and duration, BMI, resting blood pressure, and heart rate. Disease severity was categorized into six categories: mild, mild-moderate, moderate, moderate-severe, severe and very severe, based on the including physician's evaluation and patients' self-reported function level from 0 to 100% on a standardized form with scoring examples. Patients with mild or very severe ME/CFS were excluded from participation. Physical function was measured by the Short Form-36 Physical Function subscale (37, 38), and mean steps per 24 h were measured using a SenseWear® armband (BodyMedia Inc., Pittsburgh, PA, USA) in a home setting for 5–7 consecutive days (39). Demographic data (family, educational and employment status) were collected from a modified DePaul questionnaire (40) completed at baseline.

#### Biological Markers of Endothelial Dysfunction

From baseline serum samples we measured serum concentrations of amino acids and derivatives which are associated with endothelial function (41–43). These analyses were performed as part of a comprehensive metabolic profiling of participants in three ME/CFS trials (12). Potential risk markers for endothelial dysfunction and cardiovascular disease include low levels of arginine (Arg) and homoarginine (hArg) (43), and elevated levels of asymmetric dimethylarginine (ADMA) (41), symmetric dimethylarginine (SDMA) and high-sensitivity C-reactive protein (hs-CRP) (42). Arg, hArg, ADMA, and SDMA were analyzed by liquid chromatography-tandem mass spectrometry with within and between day CVs of 3–12% (44), and hs-CRP was measured by immunoMALDI mass spectrometry (45) (Bevital, Bergen, Norway).

### Statistical Analyses

Continuous variables were described with median and range, categorical with counts and percentages. Due to a limited sample size and skewed distribution, statistical comparisons were performed using non-parametric methods. Comparisons between groups (patient and reference groups) regarding baseline FMD and PORH were made using a Mann-Whitney test for independent samples. Possible associations between FMD and PORH and ME/CFS illness duration and severity and steps per 24 h were assessed with Kruskal–Wallis test. When analyzing the patients only, paired Wilcoxon signed ranks tests were used to compare FMD and PORH results at baseline and at 12 months. The difference between groups (responders and non-responders) in change of FMD/PORH values from baseline to 12 months was analyzed using a General Linear Model (GLM) of repeated measures with clinical response as a between-subjects factor. The correlation between FMD and PORH values at baseline was computed using Spearman non-parametric correlation. The result is expressed as the correlation coefficient rho. *P* < 0.05 were considered statistically significant and all tests were two-sided. We consider our study exploratory and no correction for multiple testing was performed. All analyses were done using SPSS Statistics ver. 25 (IBM Corp., Armonk, NY) and Graphpad Prism ver. 8 (Graphpad Software, La Jolla, CA).

### Ethics

The study “Cyclophosphamide in ME/CFS” including this substudy was approved by the Regional Committee for Medical and Health Research Ethics (2014/1672) in Norway. Candidates received information about the study in writing, in individual consultations with investigators (ØF, OM, IR), and in follow-up telephone consultations with the study nurse (KS). Participation was subject to signed informed consent. Ultrasound images were stored on CDs in a locked cabinet, and data registered in a secure electronic case report system (Viedoc, PCG Clinical Services, Uppsala, Sweden). All data were de-identified and the scrambling key stored in a dedicated area of the hospital's research server.

## Results

### Participant Characteristics

Fifty individuals were invited to receive information about the clinical trial. Three did not wish to participate and seven did not meet the inclusion criteria. All 40 trial participants consented to participation in the endothelial function substudy. A majority (78%) was female and young to middle aged, with a median age at baseline of 42.4 (range 21.5–61.1). All participants had an established ME/CFS diagnosis according to the Canadian diagnostic criteria. ME/CFS severity based on clinical assessment ranged from mild-moderate to severe, and self-reported physical function ranged from 5 to 40%, with a median value of 16% (range 0–100%). Half the participants reported a disease duration of more than 10 years. The median score for Short Form-36 Physical Function was 35 (raw score, range 0–100). Two patients had a medical history of cardiovascular disease (one case of myocardial infarction, one of hypertension), and 16 out of 40 reported allergies to food or other allergens. Further details concerning the demographics and clinical characteristics of the included participants are listed in Table 1.

**Table 1:** Demographics and baseline clinical characteristics of study participants.

***N* = 40**	***N***	**%**
Female sex	31	78
**Marital status/family**		
Single, widow/er, divorced	15	37.5
Married, registered partner, co-habiting	25	62.5
Have children	24	60
**Highest level of completed education**		
Primary or secondary education	17	42.5
University, college, or higher university degree	23	57.5
**Employment status**		
Work part time, homemaker	7	17.5
Work assessment allowance, disability	33	82.5
**ME/CFS severity**		
Mild-moderate	14	35
Moderate	13	32.5
Moderate-severe	7	17.5
Severe	6	15
**ME/CFS duration**		
2–5 yrs	7	17.5
5–10 yrs	13	32.5
10–15 yrs	9	22.5
>15 yrs	11	27.5
**Comorbidity**^**a**^		
Known history of cardiovascular disease	2	5
Fibromyalgia	3	7.5
Hypothyroidism	4	10
Allergy	16	40
History of depression	4	10
	**Median**	**Range**
Age, years	42.4	21.5–61.1
Body mass index, kg/m^2^	23.4	17.1–33.1
Mean steps per 24 h	2,944	568–9,637
Short form-36 physical function^b^	35	0–65
Self-reported function level, per cent^c^	16	5–40
Systolic blood pressure, mmHg	120	88–160
Diastolic blood pressure, mmHg	77	55–96
Resting heart rate, bpm	68	42–113

a *Self-reported at baseline*.

b *Raw score; range 0–100*.

c *Self-reported; range 0–100%. ME/CFS, Myalgic encephalomyelitis/chronic fatigue syndrome*.

### Endothelial Function Measurements

The entire cohort of 40 trial participants attended endothelial function tests at baseline, but not all measurements were available for all participants. Individuals with missing data at one or both time points (*n* = 6 for PORH, *n* = 13 for FMD), due to withdrawals, failure to comply with test preparations or inadequate image quality, were not included in the analyses for change from baseline to 12 months. Participants with missing data did not differ significantly with regards to age, sex, disease duration, or severity from those with complete data. See Tables 2, 3 for details.

**Table 2:** Measurements of endothelial function at baseline.

**FMD baseline median (range)**	**Healthy controls,** ***n* = 66**	**ME/CFS,** ***n* = 35**	***P*-value**
FMD, per cent	7.7 (0.7–21.0)	5.9 (0.5–13.1)	*0.005*
Arterial diameter at rest, mm	3.01 (1.61–3.80)	3.0 (2.29–4.61)	0.34
Absolute increase in mm	0.23 (0.02–0.53)	0.20 (0.02–0.33)	*0.01*
Arterial diameter after nitroglycerin^a^, mm	3.65 (2.03–4.82)	3.75 (2.90–5.80)	0.06
Increase in diameter after nitroglycerin^b^, per cent	21.2 (8.4–40.1)	25.3 (11.2–42.4)	*0.02*
FMD/nitro ratio^c^	0.37 (0.04–1.03)	0.23 (0.02–0.51)	* < 0.001*
**PORH baseline median (range)**	**Healthy controls,** ***n* = 30**	**ME/CFS,** ***n* = 39**	***P*****-value**
PORH, perfusion units	1,886 (808–8,158)	1,331 (343–4,334)	*0.003*

*ME/CFS group vs. healthy control group. Mann–Whitney U-test for independent samples*.

a *Maximum dilation after sublingual administration of nitroglycerin, absolute value*.

b *Maximum dilation after sublingual administration of nitroglycerin, increase in percent compared to baseline*.

c *Ratio of FMD (in percent) by maximum dilation after nitroglycerin (in per cent). Missing data: One patient failed to complete measurements at baseline. For FMD, a further 4 cases were excluded from analyses due to inadequate image quality. ME/CFS, Myalgic Encephalomyelitis/Chronic Fatigue Syndrome; FMD, Flow-mediated dilation; PORH, Post-occlusive reactive hyperemia. Italics denote statistically significant values*.

**Table 3:** Measurements of endothelial function, ME/CFS group at baseline vs. at 12 months.

**FMD baseline vs. 12 months median (range)**.	**ME/CFS baseline,** ***n* = 27**	**ME/CFS 12 months,** ***n* = 27**	***P*-value**
FMD, per cent	5.7 (0.5–13.1)	5.3 (0.2–15.4)	0.9
Arterial diameter at rest, mm	2.98 (2.29–4.47)	3.14 (2.38–4.61)	*0.02*
Absolute increase in mm	0.19 (0.02–0.33)	0.18 (0.01–0.48)	0.85
Arterial diameter after nitroglycerin^a^, mm	3.70 (2.90–5.30)	3.77 (2.91–5.38)	0.07
Increase in diameter after nitroglycerin^b^, per cent	25.3 (11.2–42.4)	23.5 (11.6–35.6)	0.75
FMD/nitro ratio^c^	0.23 (0.02–0.51)	0.22 (0.2–0.46)	0.75
**PORH baseline vs. 12 months median (range)**	**ME/CFS baseline,** ***n* = 34**	**ME/CFS 12 months,** ***n* = 34**	***P*****-value**
PORH, perfusion units	1,323 (343–4,334)	1,428 (387–4,335)	0.18

*Only patients with values for both timepoints included, and analyzed by Wilcoxon signed ranks test*.

a *Maximum dilation after sublingual administration of nitroglycerin, absolute value*.

b *Maximum dilation after sublingual administration of nitroglycerin, increase in percent compared to baseline*.

c *Ratio of FMD (in percent) by maximum dilation after nitroglycerin (in per cent). Missing data: 2 patients withdrew from study before 12 months, one of whom also failed to complete measurements at baseline. Four patients failed to complete measurements at 12 months, due to intercurrent illness or non-compliance with preparations. For FMD, a further 7 cases were excluded from comparative analyses due to inadequate FMD image quality at either time point. ME/CFS, Myalgic Encephalomyelitis/Chronic Fatigue Syndrome; FMD, Flow-mediated dilation; PORH, Post-occlusive reactive hyperemia. Italics denote statistically significant values*.

#### Flow-Mediated Dilation

**Figure 1:**
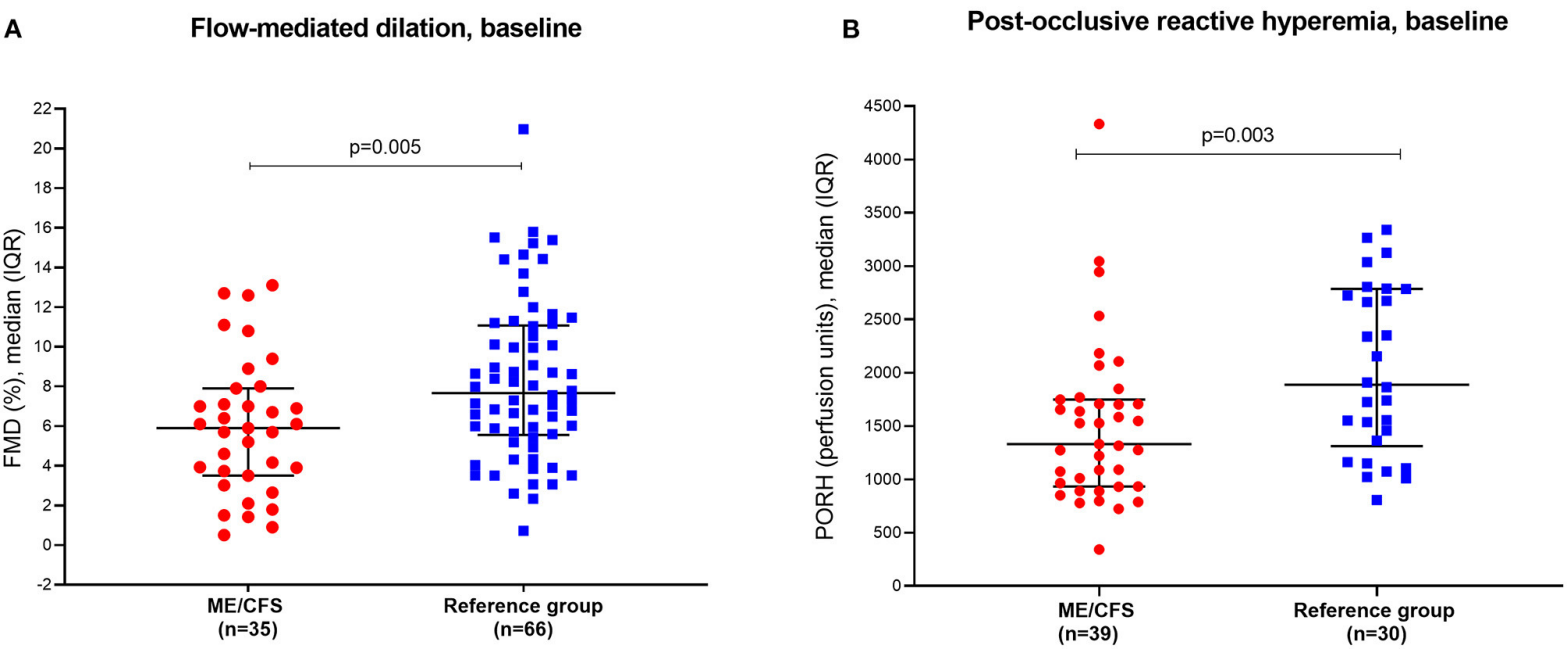
**(A)** Baseline values for flow-mediated dilation (FMD) in patients and reference group of healthy women. FMD is expressed as dilation in per cent in response to 5 min occlusion. **(B)** Baseline values for post-occlusive reactive hyperemia (PORH) in patients and reference group of healthy controls. PORH is expressed as increase in perfusion units during the first 2 min after cuff release. One healthy control outlier at 8,158 p.u. is not shown, but is included in the analysis.

At baseline, median FMD was significantly lower for ME patients compared to healthy women; 5.9 vs. 7.7%, *p* = 0.005 (Table 2, Figure 1A). FMD <5% was present in 14 (40%) of the ME patients compared to 14 (21%) of the reference group. After administration of sublingual glyceryl nitrate, maximum dilation compared to diameter at rest was higher in the ME group (25.3%) than the reference group (21.2%) (*p* = 0.02), showing intact ability to dilate vessels adequately.

#### Post-Occlusive Reactive Hyperemia

Median PORH at baseline was significantly lower for patients compared to healthy individuals; 1,331 vs. 1,886 perfusion units, *p* = 0.003 (Table 2, Figure 1B). PORH <1,000 p.u. was present in 11 (28%) of the ME group and only in one individual in the reference group (3.5%).

#### Relation to ME/CFS Severity and Duration

There were no statistically significant associations between FMD or PORH and age, BMI or ME/CFS severity measured by clinical assessment, self-reported function level or SF-36 Physical Function, disease duration, or number of steps per 24 h (Table 4).

**Table 4:** Correlations between vascular measures and laboratory and clinical parameters.

		**FMD^a^**	**PORH^b^**
		**Spearman's rho**	**Spearman's rho**
Clinical parameters	Age, years	−0.289	−0.380
	Body mass index, kg/m^2^	−0.139	−0.098
	Total function level, per cent^c^	0.009	0.128
	Short Form-36 Physical Function score^d^	−0.076	0.259
	Steps per 24 h^e^	−0.09	0.21
Laboratory parameters	Arginine, μM	0.048	0.253
	Asymmetric dimethylarginine, μM	0.022	0.050
	Symmetrical dimethylarginine, μM	−0.470^*^	0.255
	Homoarginine, μM	−0.017	0.272
	High-sensitivity C-reactive protein, μM	−0.089	0.073

a *Flow-mediated dilation at baseline*.

b *Post-occlusive reactive hyperemia at baseline*.

* p < 0.05.

c *Self-reported; range 0–100%*.

d *Raw score; range 0–100*.

e *Measured by Sensewear® armband for 5–7 consecutive days*.

#### Correlation Between FMD and PORH

There was no significant correlation between measured FMD and PORH in the patient group at baseline (*r* = −0.12, *p* = 0.47) or between changes in FMD and PORH from baseline to 12 months (*r* = 0.12, *p* = 0.53).

### Changes in Endothelial Function and Association With Symptom Change

**Figure 2:**
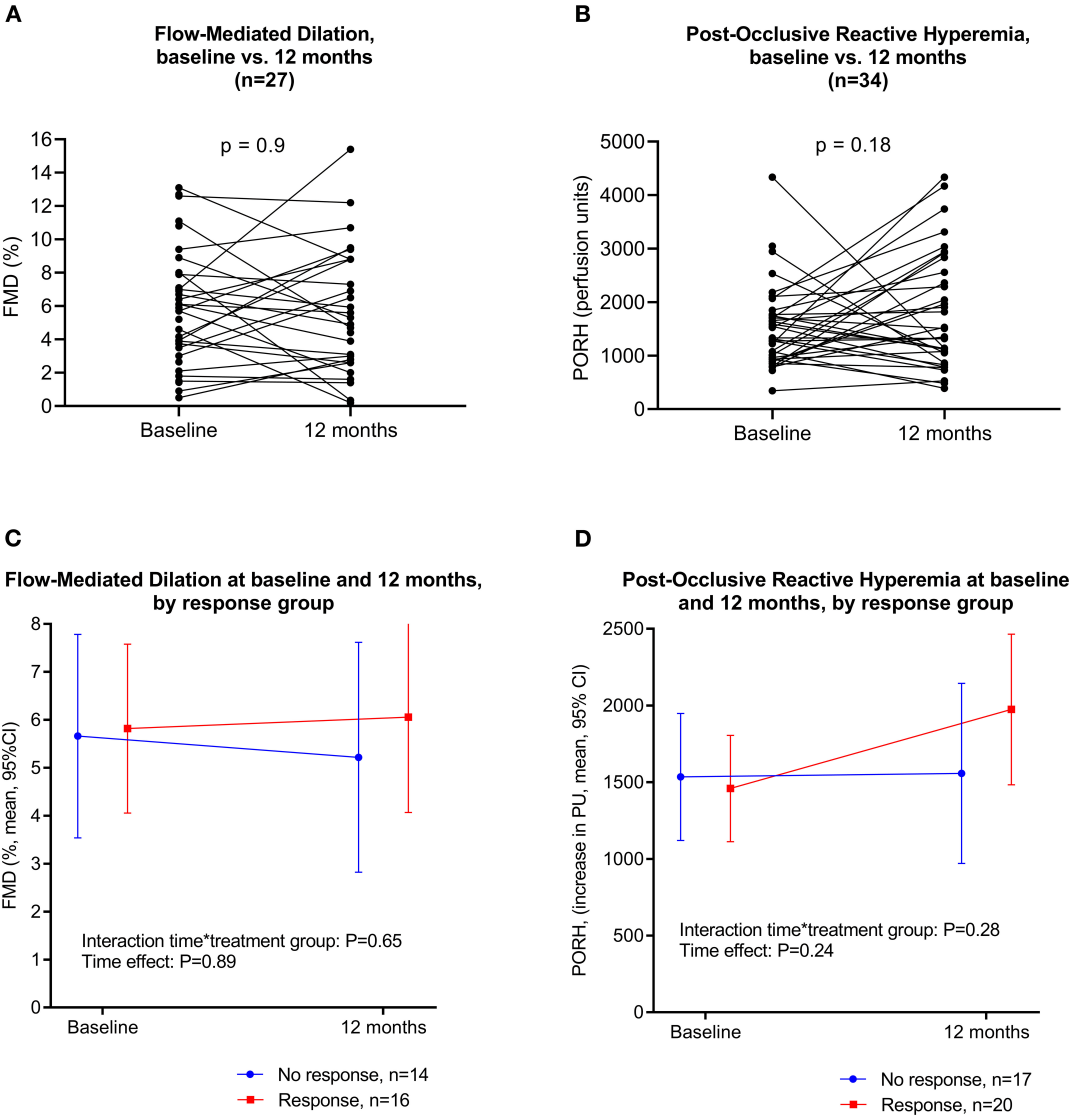
**(A)** Flow-mediated dilation at baseline and at 12 months. **(B)** Post-occlusive reactive hyperemia at baseline and at 12 months. **(C)** Flow-mediated dilation at baseline and at 12 months, by response group. GLM repeated measures with clinical response as between-subjects factor. **(D)** Post-occlusive reactive hyperemia at baseline and at 12 months, by response group. GLM repeated measures with clinical response as between-subjects factor.

Although 22 of the 40 patients reported clinical response after cyclophosphamide treatment (29), our data did not reveal any statistically significant changes in endothelial function (FMD or PORH) from baseline to 12 months (Only patients with values for both timepoints included, and analyzed by Wilcoxon signed ranks test) (Table 3, Figures 2A,B). Furthermore, GLM repeated measures analyses showed no significant differences in changes of endothelial function (FMD or PORH) from baseline to 12 months, between responders and non-responders after cyclophosphamide treatment, i.e., there were no significant differences assessed by the interaction term response group-by-time (Figures 2C,D).

### Biological Markers of Endothelial Dysfunction

There was no statistically significant difference between ME/CFS patients and healthy controls for arginine, homoarginine, ADMA, or hs-CRP. For SDMA, which is considered a risk marker for endothelial dysfunction and cardiovascular disease, the ME/CFS group had a significantly lower mean serum level than healthy controls (0.54 vs. 0.63, *p* = 0.014; Table 5). However, correlation analyses showed a significant inverse correlation between SDMA levels and FMD values, indicating a relationship between high SDMA levels and low FMD (Table 4).

**Table 5:** Serum levels of metabolites that may affect endothelial function, in ME/CFS patients and healthy controls.

**Biological markers of endothelial function. μM, median (range)**	**ME/CFS^a^** ***n* = 40**	**HC^b^** ***n* = 30**	***P*-value^c^**
Arginine	80.83 (51.88–129.81)	85.63 (52.38–136.40)	0.20
Asymmetric dimethylarginine	0.55 (0.31–0.78)	0.50 (0.34–0.69)	0.11
Symmetrical dimethylarginine	0.54 (0.38–0.78)	0.63 (0.27–0.79)	0.014^*^
Homoarginine	1.76 (0.64–3.53)	1.94 (0.90–4.24)	0.19
High-sensitivity C-reactive protein	0.35 (0.00–4.51)	0.53 (0.00–6.20)	0.38

a *ME/CFS patients, non-fasting*.

b *Healthy controls, non-fasting*.

c P-values from Mann–Whitney tests;

* *p < 0.05*.

## Discussion

This study investigated endothelial function in ME/CFS, and was the first to describe the changes in endothelial function before and after therapeutic intervention with IV cyclophosphamide in ME/CFS patients. Endothelial function measurements at baseline indicated that at the group level, the patients had significantly reduced large and small vessel endothelial function compared to healthy individuals, in line with results from previous studies (16, 17). Although more than half of the patients met the criteria for clinical response during the study, we were unable to detect any significant associations between clinical response and changes in endothelial function from baseline to 12 months follow-up.

A strength of this study was the inclusion and rigorous follow-up of well-characterized ME/CFS patients who fulfilled the Canadian consensus criteria. We used two complementary methods to measure different aspects of endothelial function. Flow-mediated dilation (FMD) is the “gold-standard” method for large vessel endothelial function assessment (21), and the measurements have followed a well-established protocol. However, FMD is a challenging and operator-dependent technique, which is subject to interpretive error. After images of suboptimal quality were excluded from analyses, inter-observer variability in this study was good to excellent. The measurements of post-occlusive reactive hyperemia (PORH) by Doppler laser are less operator-dependent, but studies show varying reproducibility (46, 47). Assessments were well-tolerated by the patients.

One weakness of the study was a possible inclusion bias. The participants were recruited for the primary purpose of a clinical, open-label drug trial, which excluded patients with either mild or very severe disease. The sample size was relatively small, but comparable to other studies using these methods. Due to the exploratory nature of the study, no power calculation or sample size assessment was performed for the purpose of the endothelial function analyses, and the study may be underpowered to detect significant correlations. The study did not include a designated control group, but relied on endothelial function data from healthy individuals included as control groups for two other studies, one of which consisted of women only. However, the median FMD values for men and women in the ME group were identical. Severity and response evaluations were largely based on physicians' assessments and patient self-report. Although patient-reported outcome measures are vital in order to capture the patient perspective, the study could have benefited from more objective parameters. By way of objective functional assessments, we performed activity monitoring using a Sensewear armband for 5–7 days at three designated time points, and we found that the patient-reported parameters correlated well with changes in activity level.

The level of physical activity of the healthy individuals was not recorded, so we have not been able to control for the effects of a sedentary or active lifestyle. A 2017 meta-analysis of the effects of exercise training on brachial artery FMD concluded that exercise training, particularly in patients with established cardiovascular disease, overweight/obesity and hypertension, contributed to a significant increase in FMD (48). However, patient inactivity alone is not a plausible main mechanism for the observed reduced endothelial dysfunction. For example, studies on vascular function in patients with rheumatoid arthritis suggested that the peripheral vasculature was adversely affected by sedentary behavior in these patients, while sedentary behavior was not predictive of endothelial dysfunction of the large vessels (49). Furthermore, we found no significant association between endothelial dysfunction and disease severity, SF-36 Physical Function scores or physical activity measured by steps per 24 h.

In our study, there was no correlation between microvascular and brachial artery endothelial function. This finding was expected, as a poor or absent correlation between FMD and PORH has also been reported in other diseases (47, 49) and in healthy individuals (46, 50). This could be explained by the different mechanisms behind the two measures. While the vasodilator nitric oxide (NO) is the principal mediator of FMD (21, 22), PORH represents a more complex response which is believed to involve metabolic vasodilators, endothelial vasodilators, sensory nerves and myogenic response to shear stress (23, 51, 52).

While endothelial function measurements are of interest on a group level, in our data there was a broad overlap between patients and controls, as well as a lack of correlation with symptom severity. Further studies and validation would certainly be required if FMD or PORH testing of ME/CFS patients were to be applied in a clinical or diagnostic setting. The lack of correlation between symptom improvement and changes in endothelial function could imply that reduced endothelial function, although present in a subgroup of ME/CFS patients, does not relate directly to the underlying pathomechanism of the disease. It is, however, also conceivable that the sample size is insufficient to detect correlations with symptom severity and improvement, such as reported by Scherbakov et al. (17).

One might speculate that endothelial dysfunction in ME/CFS could be associated with inadequate autoregulation of blood flow according to the demands of tissues, with resulting local hypoxia and lactate accumulation upon limited exertion. The clinical symptoms of ME/CFS suggest inadequate regulation of autonomic functions including blood flow. In a recent study of invasive cardiopulmonary exercise testing in upright position in ME/CFS patients, two types of peripheral neurovascular dysregulation were demonstrated; reduced cardiac output due to impaired venous return with low ventricular filling pressure (“preload failure”), and arterio-venous shunting with impaired peripheral oxygen extraction (53). These physiological changes are plausible contributors to several hallmark symptoms of ME/CFS, such as post-exertional malaise, and are associated with microcirculatory dysregulation, possibly related to small-fiber neuropathy (53).

Measures of known metabolites associated with endothelial function in cardiovascular diseases (Arg, hArg, ADMA, and SDMA) showed no significant differences between ME/CFS patients and healthy controls. This may argue for a different mechanism underlying the observed endothelial dysfunction in ME/CFS. We speculate that among ME/CFS patients, many of whom are relatively young women, the endothelial dysfunction could be related to an initial abnormal immune response, rather than atherosclerosis.

Further research is required in order to reach firm conclusions on any possible associations between ME/CFS symptoms and endothelial function. Future studies should aim to integrate objective activity measures as a supplement to validated patient-reported outcome measures, in order to control for the effect of physical activity or inactivity.

In conclusion, this study showed an association between ME/CFS and reduced endothelial function, both in large vessels assessed by FMD, and in small vessels by PORH. In this relatively small study, there were no significant associations between clinical response after cyclophosphamide and changes in FMD or PORH. Continued research efforts are warranted to further understand the possible circulatory disturbances involved in ME/CFS.

## Data Availability Statement

The raw data supporting the conclusions of this article will be made available by the authors, without undue reservation.

## Ethics Statement

The study was reviewed and approved by Regional Committee for Medical and Health Research Ethics of North Norway. The patients/participants provided their written informed consent to participate in this study.

## Author Contributions

KS, ØF, OM, and MKS: conception and design of study. KS, ØF, OM, and IR: inclusion of patients and acquisition of data. KS, MS, ØF, LR, and MCS: analysis and interpretation of data. KS: drafting of manuscript. All authors: critical revision for important intellectual content.

## Conflict of Interest

The authors declare that the research was conducted in the absence of any commercial or financial relationships that could be construed as a potential conflict of interest.

## Supplementary Material

Supplementary Material Study protocol.

## References

[1] Falk Hvidberg M Brinth LS Olesen AV Petersen KD Ehlers L. The health-related quality of life for patients with myalgic encephalomyelitis/chronic fatigue syndrome (ME/CFS). PLoS ONE. (2015) 10:e0132421. 10.1371/journal.pone.0132421 26147503 PMC4492975

[2] Nacul LC Lacerda EM Campion P Pheby D Drachler Mde L Leite JC. The functional status and well being of people with myalgic encephalomyelitis/chronic fatigue syndrome and their carers. BMC Public Health. (2011) 11:402. 10.1186/1471-2458-11-402 21619607 PMC3123211

[3] Brown AA Jason LA. Validating a measure of myalgic encephalomyelitis/chronic fatigue syndrome symptomatology. Fatigue. (2014) 2:132–52. 10.1080/21641846.2014.928014 27213118 PMC4871625

[4] Carruthers BM Jain AK De Meirleir KL Peterson DL Klimas NG Lerner AM. Myalgic encephalomyelitis/chronic fatigue syndrome. J Chron Fatigue Synd. (2003) 11:7–115. 10.1300/J092v11n01_02

[5] Nacul LC Lacerda EM Pheby D Campion P Molokhia M Fayyaz S. Prevalence of myalgic encephalomyelitis/chronic fatigue syndrome (ME/CFS) in three regions of England: a repeated cross-sectional study in primary care. BMC Med. (2011) 9:91. 10.1186/1741-7015-9-91 21794183 PMC3170215

[6] Committee on the Diagnostic Criteria for Myalgic Encephalomyelitis/Chronic Fatigue S. Beyond Myalgic Encephalomyelitis/Chronic Fatigue Syndrome: Redefining an Illness. Washington, DC: National Academies Press (2015). 25695122

[7] Missailidis D Annesley JA Fisher PR. Pathological mechanisms underlying myalgic encephalomyelitis/chronic fatigue syndrome. Diagnostics. (2019) 9:80. 10.3390/diagnostics9030080 31330791 PMC6787592

[8] Albright F Light K Light A Bateman L Cannon-Albright LA. Evidence for a heritable predisposition to chronic fatigue syndrome. BMC Neurol. (2011) 11:62. 10.1186/1471-2377-11-62 21619629 PMC3128000

[9] Schlauch KA Khaiboullina SF De Meirleir KL Rawat S Petereit J Rizvanov AA. Genome-wide association analysis identifies genetic variations in subjects with myalgic encephalomyelitis/chronic fatigue syndrome. Transl Psychiatry. (2016) 6:e730. 10.1038/tp.2015.208 26859813 PMC4872418

[10] Blomberg J Gottfries C-G Elfaitouri A Rizwan M Rosén A. Infection elicited autoimmunity and myalgic encephalomyelitis/chronic fatigue syndrome: an explanatory model. Front Immunol. (2018) 9:229. 10.3389/fimmu.2018.00229 29497420 PMC5818468

[11] Sotzny F Blanco J Capelli E Castro-Marrero J Steiner S Murovska M. Myalgic encephalomyelitis/chronic fatigue syndrome - evidence for an autoimmune disease. Autoimmun Rev. (2018) 17:601–9. 10.1016/j.autrev.2018.01.009 29635081

[12] Fluge Ø Mella O Bruland O Risa K Dyrstad SE Alme K. Metabolic profiling indicates impaired pyruvate dehydrogenase function in myalgic encephalopathy/chronic fatigue syndrome. JCI Insight. (2016) 1:e89376. 10.1172/jci.insight.89376 28018972 PMC5161229

[13] Armstrong CW McGregor NR Lewis DP Butt HL Gooley PRJM. Metabolic profiling reveals anomalous energy metabolism and oxidative stress pathways in chronic fatigue syndrome patients. Metabolomics. (2015) 11:1626–39. 10.1007/s11306-015-0816-5

[14] Naviaux RK Naviaux JC Li K Bright AT Alaynick WA Wang L. Metabolic features of chronic fatigue syndrome. (2016) 113:E5472–80. 10.1073/pnas.1607571113 27573827 PMC5027464

[15] Giloteaux L Goodrich JK Walters WA Levine SM Ley RE Hanson MR. Reduced diversity and altered composition of the gut microbiome in individuals with myalgic encephalomyelitis/chronic fatigue syndrome. Microbiome. (2016) 4:30. 10.1186/s40168-016-0171-4 27338587 PMC4918027

[16] Newton DJ Kennedy G Chan KK Lang CC Belch JJ Khan F. Large and small artery endothelial dysfunction in chronic fatigue syndrome. Int J Cardiol. (2012) 154:335–6. 10.1016/j.ijcard.2011.10.030 22078396

[17] Scherbakov N Szklarski M Hartwig J Sotzny F Lorenz S Meyer A. Peripheral endothelial dysfunction in myalgic encephalomyelitis/chronic fatigue syndrome. ESC Heart Fail. (2020) 7:1064–71. 10.1002/ehf2.12633 32154656 PMC7261521

[18] Garcia X Stein F. Nitric Oxide. Sem Pediatr Infect Dis. (2006) 17:55–7. 10.1053/j.spid.2006.04.002 16822466

[19] Tengan CH Rodrigues GS Godinho RO. Nitric oxide in skeletal muscle: role on mitochondrial biogenesis and function. Int J Mol Sci. (2012) 13:17160–84. 10.3390/ijms131217160 23242154 PMC3546744

[20] Moroni L Selmi C Angelini C Meroni PL. Evaluation of endothelial function by flow-mediated dilation: a comprehensive review in rheumatic disease. Arch Immunol Ther Exp. (2017) 65:463–75. 10.1007/s00005-017-0465-7 28361180

[21] Corretti MC Anderson TJ Benjamin EJ Celermajer D Charbonneau F Creager MA. Guidelines for the ultrasound assessment of endothelial-dependent flow-mediated vasodilation of the brachial artery: a report of the international brachial artery reactivity task force. J Am Coll Cardiol. (2002) 39:257–65. 10.1016/S0735-1097(01)01746-6 11788217

[22] Lekakis J Abraham P Balbarini A Blann A Boulanger CM Cockcroft J. Methods for evaluating endothelial function: a position statement from the European society of cardiology working group on peripheral circulation. Eur J Cardiovasc Prev Rehabil. (2011) 18:775–89. 10.1177/1741826711398179 21450600

[23] Yamamoto-Suganuma R Aso Y. Relationship between post-occlusive forearm skin reactive hyperaemia and vascular disease in patients with type 2 diabetes—a novel index for detecting micro- and macrovascular dysfunction using laser Doppler flowmetry. Diabet Med. (2009) 26:83–8. 10.1111/j.1464-5491.2008.02609.x 19125766

[24] Murdaca G Colombo BM Cagnati P Gulli R Spano F Puppo F. Endothelial dysfunction in rheumatic autoimmune diseases. Atherosclerosis. (2012) 224:309–17. 10.1016/j.atherosclerosis.2012.05.013 22673743

[25] Steyers CM III Miller FJ Jr. Endothelial dysfunction in chronic inflammatory diseases. Int J Mol Sci. (2014) 15:11324–49. 10.3390/ijms150711324 24968272 PMC4139785

[26] Cho KI Lee JH Kim SM Lee HG Kim TI. Assessment of endothelial function in patients with fibromyalgia–cardiac ultrasound study. Clin Rheumatol. (2011) 30:647–54. 10.1007/s10067-010-1599-8 20957400

[27] Moneghetti KJ Skhiri M Contrepois K Kobayashi Y Maecker H Davis M. Value of circulating cytokine profiling during submaximal exercise testing in myalgic encephalomyelitis/chronic fatigue syndrome. Sci Rep. (2018) 8:2779. 10.1038/s41598-018-20941-w 29426834 PMC5807550

[28] Fluge Ø Rekeland IG Lien K Thürmer H Borchgrevink PC Schäfer C. B-Lymphocyte depletion in patients with myalgic encephalomyelitis/chronic fatigue syndrome: a randomized, double-blind, placebo-controlled trial. Ann Inten Med. (2019) 170:585–93. 10.7326/M18-1451 30934066

[29] Rekeland IG Fosså A Lande A Ktoridou-Valen I Sørland K Holsen M. Intravenous cyclophosphamide in myalgic encephalomyelitis/chronic fatigue syndrome. An open-label phase II study. Front Med. (2020) 7:162. 10.3389/fmed.2020.00162 32411717 PMC7201056

[30] Fluge Ø Bruland O Risa K Storstein A Kristoffersen EK Sapkota D. Benefit from B-lymphocyte depletion using the anti-CD20 antibody rituximab in chronic fatigue syndrome. A double-blind and placebo-controlled study. PLoS ONE. (2011) 6:e26358. 10.1371/journal.pone.0026358 22039471 PMC3198463

[31] Fluge Ø Risa K Lunde S Alme K Rekeland IG Sapkota D. B-Lymphocyte Depletion in Myalgic Encephalopathy/ Chronic Fatigue Syndrome. An open-label phase II study with rituximab maintenance treatment. PLoS ONE. (2015) 10:e0129898. 10.1371/journal.pone.0129898 26132314 PMC4488509

[32] Sandvik MK Leirgul E Nygard O Ueland PM Berg A Svarstad E. Preeclampsia in healthy women and endothelial dysfunction 10 years later. Am J Obstet Gynecol. (2013) 209:569.e1–10. 10.1016/j.ajog.2013.07.024 23899451

[33] Koo TK Li MY. A guideline of selecting and reporting intraclass correlation coefficients for reliability research. J Chiropr Med. (2016) 15:155–63. 10.1016/j.jcm.2016.02.012 27330520 PMC4913118

[34] Hallgren KA. Computing inter-rater reliability for observational data: an overview and tutorial. Tutor Quant Methods Psychol. (2012) 8:23–34. 10.20982/tqmp.08.1.p023 22833776 PMC3402032

[35] Blacker C Greenwood DT Wesnes KA Wilson R Woodward C Howe I. Effect of galantamine hydrobromide in chronic fatigue syndrome: a randomized controlled trial. JAMA. (2004) 292:1195–204. 10.1001/jama.292.10.1195 15353532

[36] Scheibenbogen C Loebel M Freitag H Krueger A Bauer S Antelmann M. Immunoadsorption to remove ß2 adrenergic receptor antibodies in chronic fatigue syndrome CFS/ME. PLoS ONE. (2018) 13:e0193672. 10.1371/journal.pone.0193672 29543914 PMC5854315

[37] Ware JE Jr Sherbourne CD. The MOS 36-item short-form health survey (SF-36). I. Conceptual framework and item selection. Medical care. (1992) 30:473–83. 10.1097/00005650-199206000-00002 1593914

[38] Loge JH Kaasa S Hjermstad MJ Kvien TK. Translation and performance of the Norwegian SF-36 health survey in patients with rheumatoid arthritis. I. Data quality, scaling assumptions, reliability, and construct validity. J Clin Epidemiol. (1998) 51:1069–76. 10.1016/S0895-4356(98)00098-5 9817124

[39] Almeida GJ Wasko MC Jeong K Moore CG Piva SR. Physical activity measured by the SenseWear armband in women with rheumatoid arthritis. Phys Ther. (2011) 91:1367–76. 10.2522/ptj.20100291 21719635 PMC3169787

[40] Jason LA Sunnquist M. The development of the DePaul symptom questionnaire: original, expanded, brief, and pediatric versions. Front Pediatr. (2018) 6:330. 10.3389/fped.2018.00330 30460215 PMC6232226

[41] Sibal L Agarwal SC Home PD Boger RH. The role of asymmetric dimethylarginine (ADMA) in endothelial dysfunction and cardiovascular disease. Curr Cardiol Rev. (2010) 6:82–90. 10.2174/157340310791162659 21532773 PMC2892080

[42] Memon L Spasojevic-Kalimanovska V Bogavac-Stanojevic N Kotur-Stevuljevic J Simic-Ogrizovic S Giga V. Assessment of endothelial dysfunction: the role of symmetrical dimethylarginine and proinflammatory markers in chronic kidney disease and renal transplant recipients. Dis Markers. (2013) 35:173–80. 10.1155/2013/306908 24167363 PMC3774969

[43] Papageorgiou N Androulakis E Papaioannou S Antoniades C Tousoulis D. Homoarginine in the shadow of asymmetric dimethylarginine: from nitric oxide to cardiovascular disease. Amino Acids. (2015) 47:1741–50. 10.1007/s00726-015-2017-y 26123985

[44] Midttun Ø Kvalheim G Ueland PM. High-throughput, low-volume, multianalyte quantification of plasma metabolites related to one-carbon metabolism using HPLC-MS/MS. Anal Bioanal Chem. (2013) 405:2009–17. 10.1007/s00216-012-6602-6 23232958

[45] Meyer K Ueland PM. Targeted quantification of C-reactive protein and cystatin c and its variants by immuno-MALDI-MS. Anal Chem. (2014) 86:5807–14. 10.1021/ac500704y 24848523

[46] Ibrahimi K De Graaf Y Draijer R Jan Danser AH Maassen VanDenBrink A van den Meiracker AH. Reproducibility and agreement of different non-invasive methods of endothelial function assessment. Microvasc Res. (2018) 117:50–6. 10.1016/j.mvr.2018.01.004 29338981

[47] Roustit M Simmons GH Baguet J-P Carpentier P Cracowski JL. Discrepancy between simultaneous digital skin microvascular and brachial artery macrovascular post-occlusive hyperemia in systemic sclerosis. J Rheumatol. (2008) 35:1576–83. 18597404 PMC3291814

[48] Early KS Stewart A Johannsen N Lavie CJ Thomas JR Welsch M. The effects of exercise training on brachial artery flow-mediated dilation: a meta-analysis. J Cardiopulm Rehabil Prev. (2017) 37:77–89. 10.1097/HCR.0000000000000206 28033167

[49] Fenton SAM Sandoo A Metsios GS Duda JL Kitas GD Veldhuijzen van Zanten J. Sitting time is negatively related to microvascular endothelium-dependent function in rheumatoid arthritis. Microvasc Res. (2018) 117:57–60. 10.1016/j.mvr.2018.01.005 29355580

[50] Hansell J Henareh L Agewall S Norman M. Non-invasive assessment of endothelial function – relation between vasodilatory responses in skin microcirculation and brachial artery. Clin Physiol Funct Imaging. (2004) 24:317–22. 10.1111/j.1475-097X.2004.00575.x 15522039

[51] Babos L Járai Z Nemcsik J. Evaluation of microvascular reactivity with laser doppler flowmetry in chronic kidney disease. World J Nephrol. (2013) 2:77–83. 10.5527/wjn.v2.i3.77 24255889 PMC3832914

[52] Cracowski JL Roustit M. Current methods to assess human cutaneous blood flow: an updated focus on laser-based-techniques. Microcirculation. (2016) 23:337–44. 10.1111/micc.12257 26607042

[53] Joseph P Arevalo C Oliveira RKF Faria-Urbina M Felsenstein D Oaklander AL. Insights from invasive cardiopulmonary exercise testing of patients with Myalgic encephalomyelitis/chronic fatigue syndrome. Chest. (2021). 10.1016/j.chest.2021.01.082. [Epub ahead of print]. 33577778 PMC8727854

