# Supplementary material for: Reduced Endothelial Function in Myalgic Encephalomyelitis/Chronic Fatigue Syndrome–Results From Open-Label Cyclophosphamide Intervention Study
Source: Front Med (Lausanne). 2021 Mar 22;8:642710. doi: 10.3389/fmed.2021.642710 (PMC8019750; doi:10.3389/fmed.2021.642710)
Supplement: Supplementary Material — Study protocol. [file Data_Sheet_1.PDF]

ENDOTHELIAL FUNCTION IN ME/CFS

Flow-mediated Dilation (FMD)

A study has shown that ME/CFS patients have an endothelial dysfunction detected by a clinical assessment method measuring reactive vasodilation of the brachial artery after the application of a blood pressure cuff to the arm for 4-5 minutes (flow-mediated dilation, FMD). The same study showed a microvascular endothelial dysfunction measured by a test of post-occlusive reactive hyperemia (PORH) [1].
In collaboration with the department of cardiology at Haukeland University Hospital, we have performed endothelial function assessments by FMD in a total of 16 ME/CFS patients. Their mean FMD was 3.5%, and 5 out of 16 patients had an FMD of less than one per cent.
FMD in healthy women, using the same equipment and protocol in a study performed by the same two doctors, showed a mean FMD of 8.5%, and only one out of 66 healthy women had and FMD of less than two per cent. Thus, our preliminary data confirm the results from the above study [1].

Endothelial dysfunction is a risk factor for cardiovascular disease [2], and slightly to moderately reduced FMD is associated with autommune systemic diseases [3]. A slight to moderate association between FMD and depression is also described in some studies [4].
Endothelial function describes the ability of the endothelium to respond to external stimuli with local vasodilation. The main endothelium-dependent vasodilator is nitrogen oxide (NO). An increase in the blood flow through a vessel results in “shear stress” on the endothelium in the vessel walls. The shear stress affects the activity of endothelial nitrogen oxide synthase (eNOS), which in turn increases the production of NO from the substrate L-Arginine. Flow-mediated vasodilation in the brachial artery is the most widely used and validated method to measure endothelial function [5].
The preliminary data from our analyses in ME/CFS patients show a distinctly reduced FMD, and our hypothesis is that this could be a central finding which could impact on the patients’ symptoms.

Endothelial function assessments will be performed for patients included at Haukeland University Hospital for part A of the trial. FMD will be measured before intervention, and repeated during the time interval 7-9 months, with an option to repeat assessments at 11-12 months. We will record changes in endothelium-dependent dilation expressed as a change in per cent of the brachial artery diameter after a five-minute okklusjon (FMD).
We seek to answer the question if endothelial dysfunction assessed by FMD is related to the ME/CFS symptomatology or disease severity, and whether FMD changes favorably (increased FMD) in patients who experience a clinical response. FMD at baseline will be analysed for possible associations between FMD and disease severity, categorized as Mild/Moderate, Moderate, Moderate/Severe or Severe.

For participants in part B of the trial with Very Severe ME/CFS, who live near Bergen, participation in endothelial function assessments may be an option if transportation to the hospital is feasible.
Changes in FMD from baseline to 7-9 and/or11-12 months after start of intervention will be recorded, and analyses of differences between responders and non-responders may be performed.

FMD assessments are performed under standardised conditions. Guidelines for the method developed by The International Brachial Artery Reactivity Task Force [6] will be followed. Contestants should not be menstruating, and they should fast with regards to food, fluids (exept water), tobacco and medicines for 8 hours before assessment. Depot medications should not be ingested during the last 24 hours. Measurements are performed at approximately the same time of day for all participants. The assessment room is quiet and dark, with a temperature of approx. 22° C. Pre-assessment the participant relaxes in a supine position on the examination table for at least ten minutes. A blood pressure cuff is placed on the right lower arm. The ultrasound examination is performed using a GE Vingmed (GE Vingmed, Vivid E9, GE, Horten, Norway) system, with a multi-frequency linear probe, 6-13 mHz (M12L). The brachial artery is imaged in the longitudinal plane above the antecubital fossa and a baseline rest image is acquired.The measurement area is indicated on the skin using a marker pen. The blood pressure cuff, which is positioned proximally on the forearm, distally to the transducer position, is inflated to 200 mmHg or at least 50 mm Hg above systolic pressure, for 5 minutes. Following deflation of the cuff, images are recorded continuously from the same area of the artery during the next 5 minutes. The diameter of the brachial artery is measured between the insides of the endothelium on the near and far walls of the artery. All measurements are performed during end diastole. Flow mediated dilation is measured at maximal dilation, and is expressed as a percentage of the baseline diameter.
After 10 minutes rest, a dose of nitroglycerine spray (0.4 mg) is administered sublingually, and images of the brachial artery are recorded continuously for another 5 minutes. The maximal diameter is measured to assess endothelial independent vasodilation.

Microvascular endothelial function

Assessments of microvascular endothelial dysfunction will be performed for patients included at the Haukeland University Hospital, at baseline and repeated during the time interval 7-9 months after first intervention, with an option to repeat measurements after 11-12 months. The assessments will be performed at approximately the same time of day, and under the same standardised conditions as those applied to FMD assessments. Endothelial function will be estimated using a Periflux 5000 unit with laser doppler technology (Perimed, Stockholm), and we will assess post-occlusive reactive hyperaemia (PORH) in the skin [7]. PORH assessment is combined with the FMD assessment into one procedure. We will measure the skin circulation at baseline, inflate the blood pressure cuff to 200 mm Hg (or at least 50 mm Hg above systolic pressure) for 5 minutes, and finally, after cuff deflation, record the hyperaemia response during the first 2 minutes (expressed as area under the curve during 2 min. hyperaemia phase minus area under the curve during 2 min. baseline

phase).

Responders and non-responders after cyclophosphamide infusions may be compared in terms of relation between microvascular endothelial function and clinical response with symptom change. Changes in microcirculation from baseline to follow-up after 7-9 months and/or 11-12 months after start intervention are recorded and analysed for differences between responders and non-responders.

As reference values are lacking in available literature, as part of the RituxME trial we have invited 30 healthy controls between the ages of 18-65 (3/4 female, with no known chronic disease) to undergo the assessment of microvascular endothelial function with Periflux 5000 (PORH and iontophoretic application of acetylcholine). The purpose is to establish a reference material for analyses of microvascular endothelial function, and the controls will be subject to the same standardized conditions as outlined above. The controls will be recruited among staff and students at Haukeland University Hospital, and they will receive a separate information sheet and consent form. The results from this control group will also be used as reference values for patients in this substudy.

1. Newton DJ, Kennedy G, Chan KK, Lang CC, Belch JJ, et al. (2012) Large and small artery endothelial dysfunction in chronic fatigue syndrome. Int J Cardiol 154: 335-336.
2. Sitia S, Tomasoni L, Atzeni F, Ambrosio G, Cordiano C, et al. (2010) From endothelial dysfunction to atherosclerosis. Autoimmun Rev 9: 830-834.
3. Murdaca G, Colombo BM, Cagnati P, Gulli R, Spano F, et al. (2012) Endothelial dysfunction in rheumatic autoimmune diseases. Atherosclerosis 224: 309-317.
4. Cooper DC, Tomfohr LM, Milic MS, Natarajan L, Bardwell WA, et al. (2011) Depressed mood and flow-mediated dilation: a systematic review and meta-analysis. Psychosom Med 73: 360-369.
5. Sorensen KE, Celermajer DS, Spiegelhalter DJ, Georgakopoulos D, Robinson J, et al. (1995) Non-invasive measurement of human endothelium dependent arterial responses: accuracy and reproducibility. Br Heart J 74: 247-253.
6. Corretti MC, Anderson TJ, Benjamin EJ, Celermajer D, Charbonneau F, et al. (2002) Guidelines for the ultrasound assessment of endothelial-dependent flow-mediated vasodilation of the brachial artery: a report of the International Brachial Artery Reactivity Task Force. J Am Coll Cardiol 39: 257-265.
7. Roustit M, Cracowski JL (2012) Non-invasive assessment of skin microvascular function in humans: an insight into methods. Microcirculation 19: 47-64.
